# Effect of high-intensity statin preloading on TIMI flow in patients presenting with ST-elevation myocardial infarction undergoing primary percutaneous coronary intervention

**DOI:** 10.1186/s43044-020-00074-0

**Published:** 2020-07-10

**Authors:** Ahmed Shawky Elserafy, Nabil Mahmoud Farag, Ahmed Ibrahim El Desoky, Khaled Adel Eletriby

**Affiliations:** Cardiology Department, Faculty of Medicine, Ain Shams University, Abbassia square, Abbasia, Cairo 11566 Egypt

**Keywords:** High-intensity statins, Statins, STEMI, TIMI flow, MBG, Myocardial blush grade, Primary PCI

## Abstract

**Background:**

Acute ST-elevation myocardial infarction (STEMI) is a major cause of morbidity and mortality worldwide. Primary percutaneous coronary intervention (PCI) has improved the outcomes from STEMI and improved myocardial perfusion. However, there is still room for medical therapy to help perfuse the myocardium. The aim of this study was to assess the impact of high-intensity statins used prior to primary PCI in patients presenting with acute STEMI on myocardial perfusion. The study included 170 patients who presented with acute STEMI to Ain Shams University Hospitals and underwent primary percutaneous coronary intervention (PCI). They were divided into two groups where the first group received high-intensity statins (80 mg of atorvastatin or 20 mg of rosuvastatin) besides guideline-recommended therapy before primary PCI and the second group served as a control group and received guideline-recommended therapy, and high-intensity statins were given as usual after going back to the coronary care unit after primary PCI. Post-interventional thrombolysis in myocardial infarction (TIMI) flow grade and myocardial blush grade (MBG) were recorded, and ST-segment resolution was measured.

**Results:**

The LAD was the culprit vessel for the majority of patients in both groups. In the control group, there were 4 patients with TIMI I flow and MBG I, 13 with TIMI II flow and MBG II, and 68 with TIMI III flow and MBG III. Meanwhile, in the cases group, there was 1 patient with TIMI I flow and MBG I, 3 with TIMI II flow and MBG II, and 81 with TIMI III flow and MBG III. This difference was statistically significant with a *P* value of 0.010. There were 34 patients in the cases group who showed complete ST-segment resolution (40%) vs. 19 patients (22.4%) in the control group which was statistically significant with a *P* value of 0.013. In addition, ejection fraction had values of mean ± SD of 45.91 ± 5.49 in the cases group vs. 43.01 ± 8.80 in the control group which was statistically significant with a *P* value of 0.011.

**Conclusion:**

High-intensity statin loading before primary PCI resulted in improved post-procedural TIMI flow, MBG, complete ST-segment resolution, and ejection fraction.

## Background

Coronary artery disease is the most important cause of mortality in worldwide [1]. ST-elevation myocardial infarction (STEMI) is an important cause that increases morbidity and mortality too. Complications and poor outcome, including death, are not uncommon [2].

Percutaneous coronary intervention (PCI) is the most successful reperfusion strategy for flow restoration in acute coronary syndromes (ACS) [3]. Periprocedural myocardial injury and no-reflow phenomenon can still occur even though the advances in reperfusion therapy. These phenomena are associated with poor in-hospital and long-term outcomes. As no-reflow constitutes multiple mechanisms, we require various therapeutic strategies in different situations. Our weapons include the use of antiplatelet agents, vasodilators, and statins [4].

In addition to the beneficial lipid modulation effects, statins can exert a variety of pleiotropic actions. Of the inhibitions of inflammation, inhibition of ventricular remodeling improves vascular endothelial function and antioxidant effects [5]. Through the multiple mechanisms of benefit, statins have shown a significant reduction in cardiovascular morbidity and mortality both in primary and secondary preventions [6].

Multiple meta-analyses of trials have proved unquestionable proof that statins reduce the risk for acute coronary syndromes, strokes, and overall mortality in patients with established coronary heart disease as well as those without coronary heart disease but at high risk for it [7].

## Aim of the work

The aim of this study was to assess the impact of high-intensity statins used before initiation of primary PCI in patients presenting with acute STEMI on myocardial perfusion.

## Methods

Our study was conducted on 170 patients after a verbal consent presented during June 2019 to December 2019 to our university hospitals with STEMI and underwent primary PCI. Patients were divided into 2 groups (85 patients each); the first group received high-intensity statin (80 mg of atorvastatin or 20 mg of rosuvastatin) besides guideline-recommended therapy before primary PCI. The second group received guideline-recommended therapy before primary PCI.

The exclusion criteria were patients presenting with STEMI after 48 h from the onset of chest pain; those who underwent thrombolytic reperfusion therapy; those in Killip class 4; patients with hematological disorders, acute inflammatory diseases, hepatic failure, cancer, and chronic renal disease on a hemodialysis program; and patients with known allergy or intolerance to statin therapy or previously on statin therapy.

Patients were subjected to the following:

1. A detailed medical history and clinical examination to assess the inclusion criteria were done.
2. A 12-lead surface ECG at the time of diagnosis and after primary PCI to calculate the percentage of ST-segment resolution (STR). The complete early STR was defined as ≥ 70% STR [8].
3. Coronary angiography to identify their coronary anatomy, the culprit vessel causing the infarction, their TIMI flow score, and TIMI myocardial blush grade.
  TIMI flow score was assessed as follows:
  - Grade 0 (no perfusion): the absence of antegrade flow past the point of occlusion.
  - Grade 1 (penetration with no perfusion): the dye passes beyond the area of occlusion but “hangs up” and does not opacify the entirety of the coronary bed distal to the obstruction in a timely fashion.
  - Grade 2 (perfusion which is partial): the contrast material passes beyond the obstruction and opacifies the coronary bed after the obstruction. However, the rate of clearance from the distal bed (or both) is perceivably slower than that from comparable areas not perfused by the occluded vessel.
  - Grade 3 (perfusion is complete): antegrade flow into the bed past the obstruction occurs as rapidly as to proximal to the obstruction, and it clears from the involved bed as promptly as from normally perfused vessels [9].
  Myocardial blush grade (MBG) is defined as the amount of contrast opacification of the myocardium supplied by the infarct-related artery (IRA) in relation to its supplying epicardial density as seen by the operator.
  - MBG 0: there is an absence of contrast opacification of the affected myocardium.
  - MBG 1: there is a minimal opacification or persistent staining seen.
  - MBG 2: a reduced myocardial blush in the infarct area when compared to the unaffected territories.
  - MBG 3: normal opacification of the myocardium that clears promptly at the end of the washout phase, similar to unaffected territories [10].
4. Transthoracic echocardiography: routine echo study was performed which included an estimation of ejection fraction by biplane Simpson’s method by experienced operators blinded from the study protocol using a GE Vivid E95 machine

## Statistical analysis

Data were recovered, tabulated, and entered to the Statistical Package for Social Science (IBM SPSS) version 20. Qualitative data was presented as numbers and percentages, mean, standard deviations, and ranges for the quantitative data. The confidence interval was set to 95%, and the margin of error accepted was set to 5%. So, the *P* value was considered significant as follows:

*P* > 0.05 was considered non-significant (NS)

*P* < 0.05 was considered significant (S)

## Results

### Demographic data (Tables 1 and 2)

There were no statistical differences between both groups regarding age (55.89 ± 10.13 vs. 55.27 ± 10.30 years) and gender (24 females and 61 males in the control group and 29 females and 56 males in the cases group). There were no statistical differences between the 2 groups regarding smoking (65.9% in the control group and 74.1% in the cases group), hypertension (25.9% in both groups), history of ischemic heart disease (14.1% in the control group and 11.8% in the cases group), and diabetes (21.2% in the control group and 17.6% in the cases group).

**Table 1:** Comparison between cases and controls regarding age and gender

		Control, no. = 85	Cases, no. = 85	Test value	*P* value	Sig.
Age (years)	Mean ± SD	55.89 ± 10.13	55.27 ± 10.304	0.398•	0.691	NS
	Range	34–79	31–80			
Sex	Females	24 (28.2%)	29 (34.1%)	0.685*	0.408	NS
	Males	61 (71.8%)	56 (65.9%)			

*P* > 0.05: non-significant; *P* < 0.05: significant; *P* < 0.01: highly significant

•Independent *t* test

*Chi-square test

**Table 2:** Comparison between cases and controls regarding CAD risk factors

		Control		Cases		Test value*	*P* value	Sig.
		No.	%	No.	%			
Smoker	No	29	34.1	22	25.9	1.373	0.241	NS
	Yes	56	65.9	63	74.1			
DM	No	67	78.8	70	82.4	0.338	0.560	NS
	Yes	18	21.2	15	17.6			
Hypertension	No	63	74.1	63	74.1	0.000	1.000	NS
	Yes	22	25.9	22	25.9			
IHD	No	73	85.9	75	88.2	0.209	0.648	NS
	Yes	12	14.1	10	11.8			

*P* > 0.05: non-significant; *P* < 0.05: significant; *P* < 0.01: highly significant

*Chi-square test

### STEMI territory and culprit vessel (Table 3)

In Table 3, in the control group, 61 patients presented with anterior STEMI (61%) while there were 43 patients (50.6%) in the cases group which was statistically significant with a *P* value of 0.004. The control group had 16 patients with inferior STEMI (18.8%) vs. 28 patients (32.9%) in the cases group which was statistically significant with a *P* value of 0.035. There were 8 patients with posterior STEMI (9.4%) in both groups, 6 patients presented with lateral STEMI (7.1%) in only the cases group, and none in the control group with a *P* value of 0.012 which denotes statistical significance.

**Table 3:** Comparison between cases and controls regarding STEMI territory and culprit vessel

		Control		Cases		Test value*	*P* value	Sig.
		No.	%	No.	%			
STEMI	Inferior	16	18.8	28	32.9	4.416	0.035	S
	Anterior	61	71.8	43	50.6	8.024	0.004	HS
	Posterior	8	9.4	8	9.4	0.000	1.000	NS
	Lateral	0	0.0	6	7.1	6.220	0.012	S
Culprit vessel	RCA	15	17.6	26	30.6	3.889	0.048	S
	LAD	61	71.8	43	50.6	8.024	0.004	HS
	LCX	9	10.6	11	12.9	0.227	0.633	NS
	OM	0	0.0	5	5.9	5.152	0.023	S

*P* > 0.05: non-significant; *P* < 0.05: significant; *P* < 0.01: highly significant

*Chi-square test

The majority of patients in both groups had the LAD as the culprit vessel (71.8% in the control group and 50.6% in the cases group) with a statistical significance indicated by a *P* value of 0.004. The second most common culprit vessel was the RCA (17.6% in the control group and 30.6% in the cases group) which was statistically significant with a *P* value of 0.048. LCX was the culprit vessel in 10.6% of the control group patients and 12.9% of the cases group with no statistical significance. The obtuse marginal (OM) was the culprit vessel in only 5.9% of patients in the cases group and none of the control group patients which was statistically significant with a *P* value of 0.023.

### Angiographic TIMI flow score and myocardial blush grade (Table 4)

In the control group, there were 4 patients with TIMI I flow and MBG I, 13 with TIMI II flow and MBG II, and 68 with TIMI III flow and MBG III.

**Table 4:** Comparison between cases and controls regarding angiographic TIMI flow score and myocardial blush grade

		Control		Cases		Test value	*P* value	Sig.
		No.	%	No.	%			
TIMI flow	I	4	4.7	1	1.2	9.184	0.010	S
	II	13	15.3	3	3.5			
	III	68	80.0	81	95.3			
MBG	I	4	4.7	1	1.2	9.184	0.010	S
	II	13	15.3	3	3.5			
	III	68	80.0	81	95.3			

*P* > 0.05: non-significant; *P* < 0.05: significant; *P* < 0.01: highly significant

•Chi-square test

Meanwhile, in the cases group, there was 1 patient with TIMI I flow and MBG I, 3 with TIMI II flow and MBG II, and 81 with TIMI III flow and MBG III.

This difference was statistically significant with a *P* value of 0.010.

### Electrocardiography and echocardiographic parameters (Table 5)

There were 34 patients in the cases group who showed complete ST-segment resolution (40%) vs. 19 patients (22.4%) in the control group which was statistically significant with a *P* value of 0.013. In addition, ejection fraction had values of mean ± SD of 45.91 ± 5.49 in the cases group vs. 43.01 ± 8.80 in the control group which was statistically significant with a *P* value of 0.011.

**Table 5:** Comparison between cases and controls regarding electrocardiography and echocardiographic parameters

		Control, no. = 85	Cases, no. = 85	Test value	*P* value	Sig.
Complete STR	No	66 (77.6%)	51 (60.0%)	6.168*	0.013	S
	Yes	19 (22.4%)	34 (40.0%)			
Echo EF	Mean ± SD	43.01 ± 8.80	45.91 ± 5.49	− 2.573•	0.011	S
	Range	20–67	30–55			

•Independent *t* test

*Chi-square test

### In-hospital MACE (Table 6)

As shown in Table 6, there were 4 mortality cases in the control group vs. 2 in the cases group, and 2 stroke cases in the control group vs. 1 patient in the cases group.

**Table 6:** Comparison between cases and control regarding in-hospital MACE

		Control		Cases		Test value*	*P* value	Sig.
		No.	%	No.	%			
Death	No	81	95.3	83	97.6	0.691	0.406	NS
	Yes	4	4.7	2	2.4			
Stroke	No	83	97.6	84	98.8	0.339	0.560	NS
	Yes	2	2.4	1	1.2			

*Chi-square test

There was no statistical significance between the two groups regarding in-hospital death of all causes and stroke after primary PCI.

## Discussion

Angiographic no-reflow is defined as less than TIMI 3 flow or TIMI 3 flow with MBG 0 or 1 in the absence of angiographic evidence of mechanical vessel obstruction [11]. Our study tested the impact of high-intensity statin loading before primary PCI on myocardial perfusion in patients presenting with STEMI, and the main findings were as follows.

We observed a significant improvement in TIMI flow, in MBG, and also in complete ST-segment resolution, but it did not have an impact on in-hospital MACE.

In our study, in the control group, there were 4 patients with TIMI I flow and MBG I, 13 with TIMI II flow and MBG II, and 68 with TIMI III flow and MBG III.

Meanwhile, in the cases group, there was 1 patient with TIMI I flow and MBG I, 3 with TIMI II flow and MBG II, and 81 with TIMI III flow and MBG III. This difference was statistically significant with a *P* value of 0.010 indicating that the TIMI flow grade improved with high-dose statin preloading.

Our results were indistinguishable as those of the STATIN-STEMI trial, which studied 171 patients with STEMI and randomized to either 80-mg atorvastatin (*n* = 86) or 10-mg atorvastatin (*n* = 85) arms for pre-PCI treatment. MBG after primary PCI was higher in the 80-mg atorvastatin arm (MBG, 2.2 ± 0.8 vs. 1.9 ± 0.8, *P* = 0.02); the post-procedural TIMI III flow grade was higher in the 80-mg atorvastatin arm, 83, vs. the 10-mg atorvastatin arm, 76, but it was not statistically significant with a *P* value of 0.07 [12]. They also found that the corrected TIMI frame count (cTFC) was lower in the 80-mg atorvastatin arm (26.9 ± 12.3 vs. 34.1 ± 19.0, *P* = 0.01) which was not measured in our study [12].

Our results were not concordant with the NAPLES-II trial where 668 patients who were not on statin therapy were randomized to an atorvastatin 80 mg (atorvastatin group; *n* = 338) or no statin (control group; *n* = 330) the day before elective PCI, and results showed no significant difference in post-procedural TIMI flow grade (*P* value 0.68) [13]. This could be explained by the fact that in the NAPLES-II trial, the patients were undergoing elective PCI, so they do not have an acute thrombotic occlusion thus having a lower risk of no-reflow.

In our study, there was no statistical significance when comparing the two groups regarding in-hospital death of all causes and stroke after primary PCI. This is in agreement with the results of the SECURE-PCI trial in which more than four thousand patients diagnosed with acute coronary syndromes were randomized to receive 2 loading doses of 80 mg of atorvastatin (*n* = 2087) or placebo (*n* = 2104) before and a day after the PCI. For the next 30 days, all patients received 40 mg of atorvastatin. At 30 days, MACE was not reduced as 6.2% of patients in the atorvastatin group and 7.1 % in the placebo group had an adverse event (*P* = .27) [14].

Our results were not concordant with the PROVE-IT trial where 4162 patients with ACS were recruited and randomized to high-intensity statin therapy (atorvastatin, 80 mg) or standard therapy (pravastatin, 40 mg). The composite end point of death, myocardial infarction, or rehospitalization for recurrent ACS was calculated in each group at 30 days. The composite end point at 30 days occurred in 3.0% of patients receiving atorvastatin 80 mg vs. 4.2% of patients receiving pravastatin 40 mg (hazard ratio [HR] = 0.72; 95% confidence interval [CI], 0.52 to 0.99; *P* = 0.046) which shows statistical significance [15].

This was also shown in the ARMYDA-ACS trial which included 171 non-ST-segment elevation ACS patients and randomized to loading 80 mg atorvastatin (*n* = 86) or placebo (*n* = 85). All patients received 40 mg atorvastatin treatment after hospitalization. The main end point of the trial was the incidence of major adverse cardiac events (death, myocardial infarction, or unplanned revascularization) within a 30-day follow-up. Major adverse cardiac events occurred in 5% of patients in the high-dose atorvastatin arm and in 17% of those who took the placebo (*P* = 0.01) which was statistically significant [16]. This could be explained by the smaller number of patients in our study and the shorter duration of follow-up.

In our study, there were 34 patients in the cases group who showed complete ST-segment resolution (40%) vs. 19 patients (22.4%) in the control group which was statistically significant with a *P* value of 0.013.

This was similar to the results in the STATIN-STEMI trial where complete STR was significantly better in the 80-mg atorvastatin arm (34 patients [39.5%] vs. 19 patients [23.8%]; *P* = 0.03) [12].

Our study revealed that the echocardiography done the next day after primary PCI showed ejection fraction had values of mean ± SD of 45.91 ± 5.49 in the cases group vs. 43.01 ± 8.80 in the control group which was statistically significant with a *P* value of 0.011. This was not concordant with the results in the STATIN-STEMI trial where the mean LVEF was 47% in the whole patient population and there was no difference between the 2 groups [12]. This difference could be explained by the fact that in our study, the control group did not receive a statin dose before PCI while in the STATIN-STEMI trial, the control group received 10 mg of atorvastatin.

In our study, statin preloading was done using either 80 mg of atorvastatin or 20 mg of rosuvastatin in STEMI patients before undergoing primary PCI, and the control group did not receive statin preloading. This was similar to the protocol used in the STATIN-STEMI trial where the STEMI patients received 80 mg of atorvastatin before undergoing PCI, but the control group also received a statin dose before PCI in the form of 10 mg of atorvastatin [14]. In the SECURE-PCI trial, ACS patients were randomized to receive 2 loading doses of 80 mg of atorvastatin (*n* = 2087) or matching placebo (*n* = 2104) before and 24 h after a planned PCI, but only 25% of patients were presenting with STEMI [16]. In the NAPLES-II, ARMYDA-ACS, and ARMYDA-RECAPTURE trials, no STEMI patients were included in the study [13, 16, 17].

## Conclusion

High-intensity statin loading before primary PCI resulted in improved post-procedural TIMI flow, MBG, complete ST-segment resolution, and ejection fraction as measured by M-mode.

## Limitations

Single-center enrollment and a small number of cases.

Short follow-up period, thus assessment of MACE was not possible.

Other methods, besides TIMI flow and MBG, should be used in the assessment of myocardial flow such as cardiac MRI or myocardial contrast echocardiography. A larger randomized controlled study is needed to prove or disprove the results achieved in the current study.

## Data Availability

All data and equipment were available at the university.

## Abbreviations

ACS: Acute coronary syndrome
ECG: Electrocardiogram
LAD: Left anterior descending
LCX: Left circumflex
MACE: Major adverse cardiac events
MGB: Myocardial blush grade
MRI: Magnetic resonance imaging
OM: Obtuse marginal
PCI: Percutaneous coronary intervention
RCA: Right coronary artery
SD: Standard deviation
STEMI: ST-segment elevation myocardial infarction
STR: ST-segment resolution
TIMI: Thrombolysis in myocardial infarction
